# Assessing self-efficacy in type 2 diabetes management: validation of the Italian version of the Diabetes Management Self-Efficacy Scale (IT-DMSES)

**DOI:** 10.1186/s12955-018-0901-3

**Published:** 2018-04-23

**Authors:** Rossella Messina, Paola Rucci, Jackie Sturt, Tatiana Mancini, Maria Pia Fantini

**Affiliations:** 1 Department of Biomedical and Neuromotor Sciences, Section of Hygiene and Biostatistics, Alma Mater Studiorum-University of Bologna, Bologna, Italy; 2 Florence Nightingale Faculty of Nursing & Midwifery, King’s College London, London, UK; 3 Endocrine-Metabolic Disease Care Unit, Department of Internal Medicine, Istituto Sicurezza Sociale, Cailungo, San Marino

**Keywords:** Self-efficacy, Self-management, Type 2 diabetes, Psychometric properties, Questionnaire, Psychosocial aspects of diabetes, Lifestyle management, Disease management

## Abstract

**Background:**

Being highly self-efficacious is a key factor in successful chronic disease self-management. In the context of measuring self-efficacy in type 2 diabetes management, the Diabetes Management Self-Efficacy Scale (DMSES) is the most widely used scale. The aim of this study was to adapt the English version of the scale to Italian and to evaluate the psychometric properties of the Italian version of DMSES in type 2 diabetes (IT-DMSES).

**Methods:**

We conducted a cross-sectional study of people with type 2 diabetes attending the Endocrine-Metabolic Disease Care Unit of the Internal Medicine Department of San Marino State Hospital between October 2016 and February 2017.

Patients completed a socio-demographic and clinical data form, the IT-DMSES and 3 self-report questionnaires measuring diabetes distress (PAID-5), psychological well-being (WHO-5) and depression (PHQ-9).

Psychometric testing included construct validity (principal component analysis), internal consistency (Cronbach’s α coefficient) and convergent/discriminant validity (Spearman’s correlation coefficient).

Decision tree analysis was performed to classify patients into homogeneous subgroups of self-efficacy based on their demographic and clinical characteristics.

**Results:**

Participants were 110 males and 55 females, mean age of 65.2 years (SD ± 9), 56.9% had been diagnosed for 1–15 years, 63% had HbA1c level > 53 mmol/mol. Two main factors underlain the construct of self-efficacy in diabetes management: ‘Disease Management’ and “Lifestyles Management”. Disease Management had a good reliability (α = .849) and Lifestyle Management had an excellent reliability (α = .902) indicating that the instrument is internally consistent. A negative and weak correlation was found between Lifestyle management, PAID-5 (*r* = − 0.258, *p* = < 0.01) and PHQ-9 (*r* = − 0.274, *p* = < 0.01) and a positive one with WHO-5 (*r* = 0.325, *p* < 0.01) supporting convergent validity. Disease management was uncorrelated with PAID-5 (*r* = − 0.142, *p* = 0.083), PHQ-9 (*r* = − 0.145, *p* = 0.076) and weekly correlated with WHO-5 (*r* = 0.170, *p* = 0.037) confirming discriminant validity. Higher levels of self-efficacy in lifestyle management were found in patients diagnosed for at least 1 year up to 15 years and aged > 65 years and the poorest self-efficacy was found in males < 65 years.

**Conclusions:**

Results support the validity and reliability of IT-DMSES. The scale can be used in research and clinical practice to monitor type 2 diabetes self-management over time.

**Electronic supplementary material:**

The online version of this article (10.1186/s12955-018-0901-3) contains supplementary material, which is available to authorized users.

## Background

The prevalence of diabetes mellitus is increasing worldwide and it has been estimated that, by 2035, some 592 million people, one adult in 10, will have diabetes [1]. Diabetes is a major cause of blindness, kidney failure, heart attacks, stroke and lower-limb amputation [2]. Type 2 diabetes (T2D) results from the body’s ineffective use of insulin and accounts for 85% to 95% of all diabetes, and is largely the result of excess body weight and physical inactivity [1, 2].

People with diabetes have to deal with multiple tasks in order to treat and regulate their disease, and especially to prevent chronic kidney disease, central nervous system complications, damage to the blood vessels of the eye. Blood sugar control, administration of insulin or taking oral hypoglycemic drugs and life styles concerning nutrition and physical exercise are examples of daily behaviors and activities that the patient needs to plan and carry out to manage their disease. Patients indicate that they consider managing self-care activities more difficult than the diagnosis of diabetes itself [3].

The American Diabetes Association (ADA) recommends to providers that they should consider the burden of treatment and patient levels of confidence/self-efficacy for management behaviors [4].

The concept of self-efficacy originates from ‘Social Learning Theory’ and is defined as people’s beliefs in their capability to organize and execute the course of action required to deal with prospective situations [5, 6]. This description shows that people’s self-efficacy is not of a general nature, but related to specific situations and tasks, which is not the case of related concepts like self-esteem, self-confidence and locus of control [7]. Being highly self-efficacious is a key factor in successful chronic disease self-management [8, 9]. Self-efficacy, or the belief that one can self-manage one’s own health, is an important goal of health care providers, particularly in chronic illness [10].

A recent systematic review [11] identified 14 studies that conducted research in the context of measuring self-efficacy in type 2 diabetes management. The review concluded that the Diabetes Management Self-Efficacy Scale (DMSES) is the most widely used scale and also some countries such as Australia, UK and China had accepted the use of the scale as a best practiced model. The DMSES in comparison to the Diabetes Empowerment Scale [12], which assesses psychosocial self-efficacy perceptions, is focused on functional diabetes management behaviours. Moreover, it is based on self-care activities the patients have to carry out in order to manage their diabetes and to prevent complications. For this reason, the main advantage of using the DMSES is the possibility to assess attitudes regarding lifestyle, foot care, weight control, medication adherence, ability to measure blood glucose levels when necessary and also the differences between managing higher and lower blood glucose levels.

The original version of the instrument was developed in Dutch [13] and consisted of 20 items. Currently it has been validated in Greek, Korean, Chinese, Iranian, Turkish, Thai [14–19] and in an Australian [20] population, demonstrating acceptable reliability and validity. Factor analysis in Greek, Korean and Chinese versions yielded four factors, five in the Iranian version and three in the Turkish version. A UK validation study reduced the DMSES to 15 items [21]. The DMSES UK [21] was found to be negatively correlated with diabetes distress and glycated hemoglobin levels and one factor solution was found.

## Methods

### Study design and participants

The aim of this study was to adapt the English version of the DMSES to Italian and to analyse its psychometric properties. We conducted a cross-sectional study of people with type 2 diabetes attending the Endocrine-Metabolic Disease Unit Care of the Internal Medicine Department of San Marino State Hospital between October 2016 and February 2017.

A sample of 165 patients with type 2 diabetes was recruited for the full study.

Inclusion criteria were: age > 18–80 years; diagnosis of type 2 diabetes more than 6 months.

Exclusion criteria were: dementia; type 1 diabetes; gestational diabetes.

Patients attending the department are referred from general practitioners, and are patients with poor glycemic control or complications. The department provides dietary assessment and education (in group or individually), assessment and treatment in diabetic foot disease and examinations of and specialist referrals for diabetes-related complications. The nursing staff usually provides education to newly diagnosed on the insulin therapy management, hypoglycemia prevention and management and glycemic self-monitoring.

### Ethical permission

The Ethics Committee of the Institute for Social Security (ISS) of San Marino approved the study procedures (registration number: 28/2016/CERS). All eligible patients provided a written informed consent after receiving an explanation of study procedures and aims and after having an opportunity to ask questions.

### Face and content validity

The DMSES UK [21] was chosen as the most appropriate version to translate into Italian because it identified item redundancy in the instrument.

The DMSES was translated to Italian and then backtranslated to English by a bilingual English native speaker [22–25]. In order to improve the comprehensibility of the questionnaire for patients, items were reviewed by the research team, which included a public health professor, a statistician, a diabetologist and a psychologist. The translation was adapted to avoid the use of a confidential ‘you’, that is considered inappropriate when addressing an elderly person. The formal way of addressing another person in Italian implies the use of third singular (she/he). Similarly, the stem sentence was rephrased in a formal way. Specifically ‘I am confident that’ was modified to *‘To what extent you feel to be able to’*. The verb ‘to check’ was replaced with ‘to measure’ in item 1; the verb ‘to correct’ was replaced with ‘to intervene’ in items 2 and 3. The version agreed with the team was then administered by the first author to a pilot sample of 5 people with type 2 diabetes of the diabetes center using a cognitive interviewing methodology to assess the perception, usefulness and interpretation of each question of the measure [23, 24]. During completion, 5 people were asked to provide comments on items and the terminology, and comments were recorded in field notes. Results of the supervised pilot administration of the instrument indicated that patients had difficulties rating items 2, 3, 4, 7, 11 and 12 (see Table 1).

**Table 1:** Comments to items during pilot administration

Items	Comments
2, 3	(1) people who do not have access to blood glucose monitors may just have the feeling of having high or low;
	(2) people guess whether their blood glucose is low or high based on expected or unknown symptoms;
	(3) people guess how to cope with these possible symptoms by changing food intake or insulin intake;
	(4) Never experienced a low glucose so they never had to correct it.
4	“even if I am able to choose correct foods for my health, doesn’t mean that I do it because I am greedy”
7	Someone did not understand the term ‘when I am ill’, was clarified using examples like “when you have a high temperature”.
11, 12	It was necessary to explain the difference between following a healthy diet when eating outside the home (in a place that the person chooses) or eating out in places that the person does not choose (eg parties, birthdays where the person cannot choose what to eat).

The time of administration ranged from 10 to 20 min. As to items concerning ‘checking and correcting blood sugar’ or ‘adjusting the diet when increasing exercise’, if patients were not used to do these activities, they were asked to answer by imagining doing it. Self-efficacy measures the perception of self-confidence in undertaking behaviours and activities. It is therefore acceptable that, if patients did not have experience of the items, they could assess how well they might perform them. For example, they may have the knowledge to manage them well in the absence of experience. The pilot group reported that the questionnaire was interesting and introduced all the issues related to diabetes; they stated also that completing the questionnaire in the presence of a doctor may prevent people from answering the questions honestly. Following this stage, items were not further modified, and the Italian final version of DMSES (IT-DMSES) was agreed (see the Additional file 1).

### IT-DMSES

**Fig. 1:**
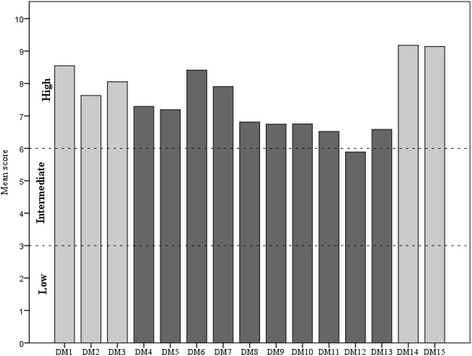
IT-DMSES items scores

The 15 items of the Italian version of DMSES measure the individual’s efficacy expectations for engaging in diabetes self-management activities, for example, checking the blood sugar, following a healthy diet even when away from home. Items are scored on a 0–10 point numerical scale, with higher scores indicating higher self-efficacy levels (Fig. 1).

### Data collection

Patients completed a socio-demographic form, 3 questionnaires and the IT-DMSES. Self-efficacy and diabetes self-management is known to be impacted by diabetes distress [26], well-being [27] and depression [28] and for this reason these outcomes were also assessed [29–31]. These assessments were used to investigate the construct validity of the IT-DMSES. When patients were unable to complete the questionnaires, they were supported by study researchers.

### The Problem Areas In Diabetes- Short form (PAID-5)

This scale measures diabetes distress, patients’ specific worries and negative emotions related to their diabetes [30, 32]. The instrument has been used in more than a hundred studies and in the DAWN MIND (monitoring individual needs in people with diabetes) [33, 34] program across ten countries. The PAID-5 short form has been validated in Italian in the BENCH-D study [35].

It includes five items with responses on a five-point Likert scale, with total score ranging from 0 to 100. A score ≥ 40 indicates elevated diabetes-related distress.

### The World Health Organization-5 Well-Being Index (WHO-5)

This scale, developed by the World Health Organization, assesses psychological well-being, a core component of quality of life [31]. The use of WHO-5 is recommended in international and some national treatment guidelines for diabetes after its worldwide use in the DAWN [36].

It includes five items with responses on a six-point Likert scale, and the total score is rescaled to range from 0 to 100. A score ≤ 50 indicates poor psychological well-being, while a score ≤ 28 indicates likely depression.

### The patient health questionnaire-9

This questionnaire is used to screen patients for a possible diagnosis of major depression. Scores range from 0 to 27, with cut-points of 5, 10, 15 and 20 indicating mild, moderate, moderately severe and severe levels of depressive symptoms [37, 38].

### Statistical analysis

The sample size was set to a minimum of 150 in order to perform an exploratory principal component analysis, for which at least a ten-to-one ratio between patients and items is recommended [39].

After descriptive analysis, principal component analysis (PCA) was performed to investigate DMSES construct validity. For this analysis, the very few missing items were replaced with mean values (28 missing items overall in 22 patients, corresponding to 1%). The number of factors to be extracted was determined according to the scree-plot method [40]. Oblique rotation was performed using the promax method, to allow for the expected correlation between factors.

Kaiser–Meyer–Olkin measure of sampling adequacy (KMO) and Bartlett’s test were calculated to evaluate the sample size adequacy. A KMO > 0.8 indicates that the sampling is adequate. The *p* value of Bartlett’s test of sphericity (which tests the null hypothesis that the original correlation matrix is an identity matrix) should be significant and lower than 0.05. Factor scores were calculated using the regression method.

Internal consistency was assessed using Cronbach’s α coefficient with cut-offs of .8 and .9 denoting good and excellent reliability.

The construct (convergent/discriminant) validity of IT-DMSES vs. the PAID-5, the WHO-5 and the PHQ-9 was analysed by using Spearman’s correlation coefficient, because of the asymmetrical frequency distribution of item responses. High levels of self-efficacy are expected to be associated with low diabetes distress, a good psychological well-being and no depressive symptoms [21, 41, 42].

Decision tree with CRT method was used to classify patients into homogeneous subgroups of self-efficacy based on demographic and clinical characteristics, including gender, age, years of education and duration of illness. All analyses were performed using IBM SPSS, version 20.

## Results

### Patient characteristics

The study sample consists of 165 patients. Participants had a mean age of 65.2 (SD ± 9) years, 56.9% had been diagnosed for 1 to 15 years, 63% reported HbA1c levels > 53 mmol/mol, 66.7% were males, 79.7% were living with a spouse or partner and 71.5% were retired. Other clinical characteristics are reported in Table 2.

**Table 2:** Demographic and clinical characteristics of study participants (*N* = 165) and scales measuring self-efficacy, depression, diabetes distress and well-being

Characteristics	N(%) or mean ± SD
Gender	
Males	110 (66.7%)
Females	55 (33.3%)
Age (years) (mean ± SD)	65.2 ± 9 (range 35–80)
Living situation	
With a spouse/partner	114 (79.7%)
With parents	7 (4.9)
Alone	22 (15.4%)
Level of education	
Elementary school	54 (33.1%)
Middle school	63 (38.7%)
High school	34 (20.9%)
College and above	12 (7.4%)
No. of years since diagnosed with diabetes, no. (%)	
< 1 year	11 (6.9%)
1–15 years	91 (56.9%)
> 15 years	58 (36.3%)
Occupational status	
Employed	40 (24.2%)
Retired	118 (71.5%)
Unemployed	2 (1.2%)
BMI (kg/m^2^)	
Underweight	1 (0.6%)
Normal weight	20 (12.1%)
Overweight	51 (30.9%)
Obese	94 (57.0%)
BMI 30–34.99	55 (33.3%)
BMI 35–39.99	27 (16.4%)
BMI ≥ 40	10 (6.1%)
HbA1c^a^ (mean ± SD)	57.28 ± 10.3
HbA1c ≤53 mmol/mol	61 (37%)
HbA1c > 53 mmol/mol	104 (63%)
Treatment regimen	
Diet/exercise only	10 (6.1%)
Oral hypoglycemic agent	71 (43%)
Insulin	11 (6.7%)
Oral hypoglycemic agent **+** insulin	73 (44.2%)
Co-morbidities	
Hypertension	126 (76.4%)
Thyroid disease	45 (27.3%)
Dyslipidemia	141 (85.5%)
Ischemic heart disease	33 (20%)
Complications	
Kidney disease	23 (13.9%)
Eye damage	26 (15.8%)
Neurological disease	21 (12.7%)
Foot complications	2 (1.2%)
Peripheral circulatory complications	12 (7.3%)
IT-DMSES scores^b^	
Mean IT-DMSES 1 factor score	8.53 ± 1.63
Mean IT-DMSES 2 factor score	6.83 ± 1.76
PHQ-9 score^c^	
No depression	99 (62.7%)
Mild depression	40 (25.2%)
Moderate depression	17 (10.8%)
Moderately severe depression	2 (1.3%)
Mean PAID-5 score^d^	39.32 ± 27.14
Cut-off ≥40 (elevated diabetes distress)	83 (51.2%)
Mean WHO-5 score^e^	63.43 ± 21.21
Good psychological well-being	118 (74.2%)
Poor psychological well-being	28 (17.6%)
Likely depression	13 (8.2%)

*Abbreviations: IT-DMSES* Italian version of the Diabetes Management Self-Efficacy Scale, *PHQ-9* Patient Health Questionnaire, *PAID-5* the Problem Areas in Diabetes-Short Form, *WHO-5* Well-Being Index

^a^HbA1c values: generic target, not modified on patient characteristics

Missing values: 22 living situation; 2 level of education; 5 n. of years since diagnosed with diabetes; 5 occupational status;

^b^6 missing values

^c^7 missing values

^d^3 missing values

^e^6 missing values

PHQ-9 scores indicated that 62.7% of patients had no depressive symptoms, 25.2% mild, 10.8% moderate and 1.3% moderate to severe depressive symptoms. PAID-5 scores showed that 51.2% of patients had elevated diabetes distress. WHO-5 scores indicated that 74.2% of patients had good psychological well-being, 17.6% had poor psychological well-being and 8.2% likely depression.

### Principal component analysis

Patients who completed the IT-DMSES were included in all the analyses (*N* = 159). The KMO index was 0.86, indicating that the sample was adequate for factor analysis and Bartlett’s test of sphericity was significant, indicating strong correlation between variables. The PCA extracted three factors that accounted for 66.8% of the total variance. However, one of the factors included only two items and its eigenvalue was marginally higher than unity. Thus, a two-factor solution was tried that was easily interpretable, and accounted for 56.6% of item variance. Factor 1 (including items: 1, 2, 3, 6, 14, 15) was labeled as “disease management” and factor 2 (including items: 4, 5, 7, 8, 9, 10, 11, 12, 13) was labeled as “lifestyles management”. Table 3 shows the item loadings on the two factors. Two items had a cross-loading (item n. 6, factor 1 = .415 factor 2 = .322; item n.7, factor 1 = .359 factor 2 = .444). Disease Management had a good reliability (α = .849) and Lifestyle Management had an excellent reliability (α = .900).

**Table 3:** Factor loadings of the two factors extracted using principal component analysis with promax rotation

	Factor 1 Disease management	Factor 2 Lifestyle management
1.check my blood sugar where necessary	.747	
2.correct my blood sugar when the sugar level is too high	.731	
3.correct my blood sugar when the blood sugar level is too low	.789	
4.choose the correct foods		.714
5.keep my weight under control		.732
6.examine my feet for cuts	.415	.322
7.adjust my eating plan when ill	.359	.444
8.follow a healthy eating pattern most of the time		.826
9.take more exercise if the doctor advises me to		.753
10.when taking more exercise I am able to adjust my eating plan		.573
11.follow a healthy eating pattern when I am away from home		.865
12.follow a healthy eating pattern when I am eating out or at a party		.844
13.adjust my eating plan when I am feeling stressed or anxious		.638
14.take my medication as prescribed	.814	
15.adjust my medication when I am ill	.870	

### Convergent/discriminant validity

Patients who completed all scales were included in this analysis (*N* = 151).

A negative and weak correlation was found between DMSES factor 2 (Lifestyle management), PAID-5 (*r* = − 0.258, p = < 0.01) and PHQ-9 (*r* = − 0.274, p = < 0.01) and a positive one with WHO-5 (*r* = 0.325, *p* < 0.01) supporting convergent validity. This suggests that patients with higher self-efficacy had a higher well-being, lower distress and fewer depressive symptoms.

DMSES factor 1 (Disease management) was uncorrelated with PAID-5 (*r* = − 0.142, *p* = 0.083), PHQ-9 (*r* = − 0.145, *p* = 0.076) and weekly correlated with WHO-5 (*r* = 0.170, *p* = 0.037) confirming discriminant validity.

### Decision tree analysis

**Fig. 2:**
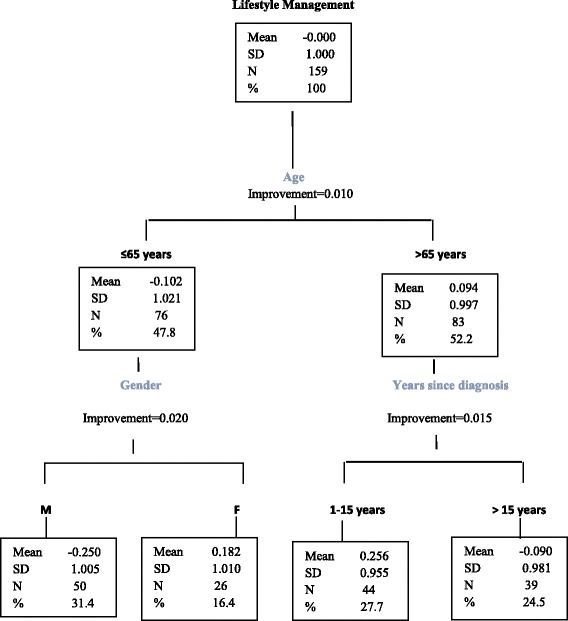
Decision tree analysis

Decision tree analysis conducted on the second DMSES factor generated four nodes (Fig. 2): lifestyle management was best among people > 65 years diagnosed for 1–15 years (0.256 ± 0.955) followed by women younger than 65 years (mean ± SD 0.182 ± 1.010), people > 65 years diagnosed for more than 15 years (− 0.090 ± 0.981) and it was worst among males younger than 65 years (− 0.250 ± 1.005). On the contrary, decision tree analysis of the disease management factor did not allow to split the sample into homogeneous sub-groups.

### Scoring instructions

Since IT-DMSES consists of two factors, two scores are necessary. Score ‘Disease Management’ is the weighted mean of items 1, 2, 3, 6, 14, 15. Score ‘Lifestyle Management’ is the weighted mean of items 4, 5, 7, 8, 9, 10, 11, 12, 13. Both of them range from 0 to 10: 0–3 denotes low levels of self-efficacy, 4–6 intermediate levels of self-efficacy, 7–10 high levels of self-efficacy. Weights are provided in Table 4.

**Table 4:** Weights of the all items for the IT-DMSES scoring

	Weights	
	Component	
	lifestyle	disease
DM1	−.006	.211
DM2	.024	.206
DM3	.006	.222
DM4	.146	.025
DM5	.151	−.021
DM6	.064	.116
DM7	.090	.099
DM8	.170	−.004
DM9	.155	−.019
DM10	.117	.046
DM11	.179	−.045
DM12	.174	−.047
DM13	.131	.035
DM14	−.031	.230
DM15	−.040	.246

## Discussion

The objective of this study was to validate the Italian version of the Diabetes Management Self-Efficacy Scale in patients with type 2 diabetes.

The study suggested that IT-DMSES is not unidimensional, and two main factors underlie the construct of self-efficacy in diabetes management. This two-factor solution explains 56.6% of items variance, demonstrating reliability of the self-efficacy construct. The first factor was clearly interpretable as ‘Disease Management‘, as it included items encompassing behaviors related to self-glucose monitoring (e.g. I am able to correct my blood sugar when the blood sugar level is too low) and medication adherence (e.g. I am able to take my medication as prescribed). The second factor was named ‘Lifestyle Management’ because it explores lifestyle interventions (e.g. I am able to choose the correct foods; I am able to take more exercise if the doctor advises me to).

Notably, Sturt et al. [21], in the validation study of the English version of DMSES scale found a one-factor solution. A possible interpretation of the discrepancies in the factor solutions is that sample characteristics differ between studies. While in the English validation [21] the patient population was recruited in a primary care setting, our sample consists of older tertiary care patients with higher levels of diabetes distress.

Our results show that the DMSES factor 2 ‘Lifestyle Management’ has a good convergent validity with the Well-Being index, suggesting that a higher perceived capability to manage diet and exercise is associated with subjective psychological well-being. This result is consistent with previous studies, in which higher self-efficacy was related to lower emotional distress [21, 43]. Factor 1 ‘Disease Management’ was uncorrelated with PAID-5, PHQ-9 and WHO-5, confirming that this factor measures a conceptually different construct from distress, depression and well-being.

The identification of two dimensions of self-confidence in diabetes management has important implications on targeting personalised patient education interventions because it allows to know the activities in which patients are facing more difficulties.

In addition, we found that self-efficacy is related to illness duration, gender and age. Higher levels of self-efficacy in lifestyle management were found in patients diagnosed for at least 1 year up to 15 years and aged > 65 years and the poorest self-efficacy was found in males < 65 years. A possible explanation for the higher self-efficacy in lifestyle management among people diagnosed for up to 15 years as opposed to those diagnosed for a longer time is that the former may tend to adhere more strictly to the recommendations of the clinical team in order to prevent complications. Concerning the low self-efficacy in men aged < 65 years as opposed to women with the same age, a possible explanation is that in Italy the choice and the preparation of food is usually a women’s task, and men may feel less efficacious in performing activities in which they are usually not involved.

These results suggest that efforts to promote patient education to self-efficacy should be especially targeted to younger man, and to patients with a long-standing experience of disease.

The study has some limitations, one of which is the external validity, in fact the study sample attending the diabetes center included mostly elderly patients with comorbid diseases and complications. Therefore, our results cannot be generalized to all patients with type 2 diabetes.

However, in order to assess the extent to which this limitation affects our results, we have analysed the correlation of disease management and lifestyle management with age, the number of complications and the number of comorbidities. These analyses indicate that correlations are close to zero, thereby mitigating this limitation.

Another possible limitation is the social desirability bias, that is the tendency to over-report good behaviors when answering questions. This may leads to an overestimation of patients’ ability to manage their diabetes. In the Iranian validation study this result was interpreted in terms of high personal expectations of patients on their ability to initiate and comply with diabetes self-management [17] and in the Australian DMSES validation study a selection bias towards motivated and self-effective patients may account for high scores [20].

## Conclusions

Our data highlight that the IT-DMSES version has sound psychometric properties and measures two different dimensions of self-efficacy: disease and lifestyle management. Results support the validity and reliability of the instrument. IT-DMSES can be used in research and clinical practice in people living with type 2 diabetes to monitor diabetes self-management over time.

## Additional file

Additional file 1: IT-DMSES and DMSES UK. (DOCX 24 kb)

## Abbreviations

ADA: American Diabetes Association
CRT: Classification and Regression Trees statistical method
DMSES: Diabetes Management self-efficacy scale
HbA1c: Glycated Hemoglobin
ISS: Institute for Social Security of San Marino
IT-DMSES: The Italian version of the Diabetes Management Self-Efficacy Scale
KMO: Kaiser-Meyer-Olkin measure
PAID-5: The Problem Areas In Diabetes-Short form
PCA: Principal Component Analysis
PHQ-9: The Patient Health Questionnaire
T2D: Type 2 diabetes
WHO-5: The World Health Organization-5 Well Being Index
