# Supplementary material for: Assessing self-efficacy in type 2 diabetes management: validation of the Italian version of the Diabetes Management Self-Efficacy Scale (IT-DMSES)
Source: Health Qual Life Outcomes. 2018 Apr 23;16:71. doi: 10.1186/s12955-018-0901-3 (PMC5914030; doi:10.1186/s12955-018-0901-3)
Supplement: Supplementary file 1 — IT-DMSES and DMSES UK. (DOCX 24 kb) [file 12955_2018_901_MOESM1_ESM.docx]

**Scala di autoefficacia nella gestione del diabete (IT-DMSES)**

***Istruzioni:***

Sotto sono indicate una serie di attività da mettere in atto per gestire il diabete. Segni con una **X** il numero che meglio descrive quanto lei ritiene di essere in grado di fare queste attività su una scala da 0 a 10. Per esempio, se crede di essere completamente capace di controllare la glicemia quando necessario, metta una X sul numero 10.

**Quanto ritiene di essere in grado di…**

| Non sono  in grado | | | | | | | Sono abbastanza  in grado | | | | | Sono completamente  in grado | | | | | | | |
| --- | --- | --- | --- | --- | --- | --- | --- | --- | --- | --- | --- | --- | --- | --- | --- | --- | --- | --- | --- |
| 1. Misurare la sua glicemia quando necessario | | | | | | | | | | | | | | | | | | | |
| 0 | 1 | 2 | 3 | | 4 | | | 5 | | | 6 | | 7 | | 8 | | 9 | | 10 |
| 1. Intervenire sulla sua glicemia quando è troppo alta   (ad esempio mangiando cibi differenti) | | | | | | | | | | | | | | | | | | | |
| 0 | 1 | 2 | 3 | | 4 | | | 5 | | | 6 | | 7 | | 8 | | 9 | | 10 |
| 1. Intervenire sulla sua glicemia quando è troppo bassa   (ad esempio mangiando cibi differenti) | | | | | | | | | | | | | | | | | | | |
| 0 | 1 | 2 | 3 | | 4 | | | 5 | | | 6 | | 7 | | 8 | | 9 | | 10 |
| 1. Scegliere gli alimenti corretti per la sua salute | | | | | | | | | | | | | | | | | | | |
| 0 | 1 | 2 | 3 | | 4 | | | 5 | | | 6 | | 7 | | 8 | | 9 | | 10 |
| 1. Tenere sotto controllo il suo peso | | | | | | | | | | | | | | | | | | | |
| 0 | 1 | 2 | | 3 | | 4 | | | 5 | 6 | | 7 | | 8 | | 9 | | 10 | |
| 1. Esaminare i suoi piedi   (ad esempio verificare la presenza di lesioni o ulcere) | | | | | | | | | | | | | | | | | | | |
| 0 | 1 | 2 | 3 | | 4 | | | 5 | | | 6 | | 7 | | 8 | | 9 | | 10 |
| 1. Correggere la sua alimentazione quando è malato | | | | | | | | | | | | | | | | | | | |
| 0 | 1 | 2 | 3 | | 4 | | | 5 | | | 6 | | 7 | | 8 | | 9 | | 10 |

**Quanto ritiene di essere in grado di…**

| Non sono  in grado | | | | | Sono abbastanza  in grado | | | Sono completamente  in grado | | | | |
| --- | --- | --- | --- | --- | --- | --- | --- | --- | --- | --- | --- | --- |
| 1. Seguire solitamente una corretta alimentazione | | | | | | | | | | | | |
| 0 | 1 | 2 | 3 | 4 | | 5 | 6 | | 7 | 8 | 9 | 10 |
| 1. Fare più esercizio fisico se il medico glielo suggerisce | | | | | | | | | | | | |
| 0 | 1 | 2 | 3 | 4 | | 5 | 6 | | 7 | 8 | 9 | 10 |
| 1. Modificare la sua alimentazione se svolge più attività fisica del solito | | | | | | | | | | | | |
| 0 | 1 | 2 | 3 | 4 | | 5 | 6 | | 7 | 8 | 9 | 10 |
| 1. Seguire un’alimentazione sana anche quando è fuori casa | | | | | | | | | | | | |
| 0 | 1 | 2 | 3 | 4 | | 5 | 6 | | 7 | 8 | 9 | 10 |
| 1. Seguire un’alimentazione sana quando mangia fuori   (ad esempio quando mangia al ristorante o ad una festa) | | | | | | | | | | | | |
| 0 | 1 | 2 | 3 | 4 | | 5 | 6 | | 7 | 8 | 9 | 10 |
| 1. Mantenere il piano alimentare quando si sente stressato o ansioso | | | | | | | | | | | | |
| 0 | 1 | 2 | 3 | 4 | | 5 | 6 | | 7 | 8 | 9 | 10 |
| 1. Assumere la terapia come da prescrizione medica | | | | | | | | | | | | |
| 0 | 1 | 2 | 3 | 4 | | 5 | 6 | | 7 | 8 | 9 | 10 |
| 1. Mantenere la sua terapia anche quando è malato | | | | | | | | | | | | |
| 0 | 1 | 2 | 3 | 4 | | 5 | 6 | | 7 | 8 | 9 | 10 |

**Self-Efficacy (or confidence) Questionnaire For People Living With Type 2 Diabetes.**

**Directions**

Below is a list of activities you have to perform to manage your diabetes. Please read each one and then put a line [**/**] through the number which best describes how **confident** you usually are that you could carry out that activity.

For example, if you are completely confident that you are able to check your blood sugar levels when nessessary, put a line through 10. If you feel that most of the time you could not do it, put a line through 1 or 2.

**I am confident that……..**

| Cannot do  At all | | | | | | | | | Maybe yes  Maybe no | | | | | | | Certain  can do | | | | | | |
| --- | --- | --- | --- | --- | --- | --- | --- | --- | --- | --- | --- | --- | --- | --- | --- | --- | --- | --- | --- | --- | --- | --- |
| 1 | I am able to check my blood sugar if necessary | | | | | | | | | | | | | | | | | | | | | |
|  | 0 | 1 | | 2 | | 3 | | 4 | | | 5 | | 6 | | 7 | | | 8 | | 9 | | 10 |
| 2 | I am able to correct my blood sugar when the sugar level is too high | | | | | | | | | | | | | | | | | | | | | |
|  | 0 | 1 | | 2 | | 3 | | 4 | | | 5 | | 6 | | 7 | | | 8 | | 9 | | 10 |
| 3 | I am able to correct my blood sugar when the blood sugar level is too low | | | | | | | | | | | | | | | | | | | | | |
|  | 0 | 1 | | 2 | | 3 | | 4 | | | 5 | | 6 | | 7 | | | 8 | | 9 | | 10 |
| 4 | I am able to choose the correct foods | | | | | | | | | | | | | | | | | | | | | |
|  | 0 | 1 | | 2 | | 3 | | 4 | | | 5 | | 6 | | 7 | | | 8 | | 9 | | 10 |
| 5 | I am able to keep my weight under control | | | | | | | | | | | | | | | | | | | | | |
|  | 0 | 1 | 2 | | 3 | | 4 | | | 5 | | 6 | | 7 | | | 8 | | 9 | | 10 | |
| 6 | I am able to examine my feet for cuts | | | | | | | | | | | | | | | | | | | | | |
|  | 0 | 1 | | 2 | | 3 | | 4 | | | 5 | | 6 | | 7 | | | 8 | | 9 | | 10 |
| 7 | I am able to adjust my eating plan when ill | | | | | | | | | | | | | | | | | | | | | |
|  | 0 | 1 | | 2 | | 3 | | 4 | | | 5 | | 6 | | 7 | | | 8 | | 9 | | 10 |

**I am confident that……..**

| 8 | I am able to follow a healthy eating pattern most of the time | | | | | | | | | | |
| --- | --- | --- | --- | --- | --- | --- | --- | --- | --- | --- | --- |
|  | 0 | 1 | 2 | 3 | 4 | 5 | 6 | 7 | 8 | 9 | 10 |
| 9 | I am able to take more exercise if the doctor advises me to | | | | | | | | | | |
|  | 0 | 1 | 2 | 3 | 4 | 5 | 6 | 7 | 8 | 9 | 10 |
| 10 | When taking more exercise I am able to adjust my eating plan | | | | | | | | | | |
|  | 0 | 1 | 2 | 3 | 4 | 5 | 6 | 7 | 8 | 9 | 10 |
| 11 | I am able to follow a healthy eating pattern when I am away from home | | | | | | | | | | |
|  | 0 | 1 | 2 | 3 | 4 | 5 | 6 | 7 | 8 | 9 | 10 |
| 12 | I am able to follow a healthy eating pattern when I am eating out or at a party | | | | | | | | | | |
|  | 0 | 1 | 2 | 3 | 4 | 5 | 6 | 7 | 8 | 9 | 10 |
| 13 | I am able to adjust my eating plan when I am feeling stressed or anxious | | | | | | | | | | |
|  | 0 | 1 | 2 | 3 | 4 | 5 | 6 | 7 | 8 | 9 | 10 |
| 14 | I am able to take my medication as prescribed | | | | | | | | | | |
|  | 0 | 1 | 2 | 3 | 4 | 5 | 6 | 7 | 8 | 9 | 10 |
| 15 | I am able to adjust my medication when I am ill | | | | | | | | | | |
|  | 0 | 1 | 2 | 3 | 4 | 5 | 6 | 7 | 8 | 9 | 10 |

The English version of the Diabetes Management Self-Efficacy Scale (DMSES UK) was developed by:

Prof. Jackie Sturt (e-mail: [jackie.sturt@kcl.ac.uk](mailto:jackie.sturt@kcl.ac.uk))

The original version of the *'Diabetes Management Self-Efficacy Scale for Type 2 Diabetes'* is developed by:

Dr. Jaap van der Bijl (e-mail: [Jaap.vanderBijl@inholland.nl](mailto:Jaap.vanderBijl@inholland.nl))

Prof. dr. Lillie Shortridge-Baggett (e-mail: [lshortridgebaggett@pace.edu](mailto:lshortridgebaggett@pace.edu))

**References:**

Sturt J, Heasrnshaw H, Wakelin M. Validity and reliability of the DMSES UK: a measure of self-efficacy for type 2 diabetes. Primary Health Care Research & Development. 2010. doi:10.1017/S1463423610000101.

Bijl J. van der, Poelgeest-Eeltink A van, & Shortridge-Baggett L.M. The psychometric properties of the diabetes management self-efficacy scale for patients with Type 2 diabetes mellitus.  *Journal of Advanced Nursing. 1999; 30*(2):352-359.
